# Occupational Therapy Practice With Cognitive Behavioral Therapy for Early Postoperative Pain: A Case Report

**DOI:** 10.7759/cureus.45882

**Published:** 2023-09-24

**Authors:** Ryusei Hara, Yuki Hiraga, Yoshiyuki Hirakawa

**Affiliations:** 1 Department of Health Care Administration and Management, Graduate School of Kyushu University, Fukuoka, JPN; 2 Department of Rehabilitation, Fukuoka Rehabilitation Hospital, Fukuoka, JPN; 3 Department of Health Sciences, International University of Health and Welfare Graduate School, Fukuoka, JPN; 4 Department of Occupational Therapy, International University of Health and Welfare, Fukuoka, JPN

**Keywords:** psychological factors, occupational therapy, high tibial osteotomy, cognitive behavioral therapy, chronic pain

## Abstract

Structuring psychological interventions using cognitive behavioral therapy in the early postoperative period is crucial to mitigate the risk of chronic pain. In this study, specific practices were presented through the case of a woman in her 60s who experienced psychological factors, such as catastrophic thinking and anxiety, due to pain after high tibial osteotomy. The aim was to evaluate the structure of cognitive behavioral therapy and its application in the early postoperative period. Cognitive behavioral therapy was initiated postoperatively, incorporating three phases: cognitive restructuring, coping skills and active pacing, and occupational therapy. As a result of occupational therapy using cognitive behavioral therapy, the patient’s pain and anxiety are reduced while achieving the goals. This case study suggests that cognitive behavioral therapy may promote goal attainment in cases where postoperative pain exacerbates psychological factors, such as catastrophic thinking and anxiety, ultimately leading to functional impairments. Implementation of cognitive behavioral therapy in Japan is lacking, necessitating urgent development. This case report serves as a foundational step in structuring cognitive behavioral therapy during the early postoperative period in Japan.

## Introduction

Chronic pain is characterized as an unpleasant sensory and emotional experience associated with, or resembling that associated with, actual or potential tissue damage [1]. Patients with chronic pain have been known to experience a diminished quality of life (QOL) and psychological anguish [2]. In Japan, the prevalence of chronic pain is 15.4% [3]. Psychosocial elements, such as catastrophic ideation, anxiety, and depression, have been identified as contributing factors to chronic pain [4]. The Japanese guidelines for chronic pain advocate for the use of cognitive-behavioral therapy (CBT) as a psychological intervention for chronic pain [5]. These guidelines acknowledge that the implementation system for CBT in Japan is inadequate, necessitating urgent development [5]. Hence, advocating for the systematization of CBT for chronic pain in Japan is imperative. A prior Japanese study demonstrated that CBT interventions for patients with chronic pain significantly impacted social QOL and psychological aspects, signifying the suitability of CBT as a treatment modality for chronic pain in Japan [6]. Conversely, in musculoskeletal diseases, pain during the initial postoperative phase acts as a mediator for psychological factors, influencing long-term postoperative well-being [7]. While orthopedic treatments, such as high tibial osteotomy (HTO) for knee osteoarthritis, have shown potential in improving QOL while preserving joint integrity, patients who experience preoperative psychological distress may encounter difficulties in resuming societal roles, including a return to work [8]. Thus, it is crucial to examine the effectiveness of CBT-based treatment methodologies during the early postoperative period since it is in this phase that patients facing pain and psychosocial factors encounter increased vulnerability to developing chronic pain. The American Occupational Therapy (OT) Association recognizes the significance of OT for pain, emphasizing individualized intervention focused on non-pharmacological self-management and promoting functional and meaningful participation in life [9]. In Japan, where manual and folk therapies are prevalent, the introduction of this approach is crucial in managing patients with chronic pain [3]. In Japan, CBT's efficacy in addressing chronic pain has been previously documented [6]. However, there remains a dearth of comprehensive reports on CBT interventions and methodologies specifically designed for patients experiencing early postoperative pain who are at a heightened risk of developing chronic pain. Therefore, the present study sought to introduce a CBT intervention for early postoperative pain in patients undergoing HTO through a representative case study. In this case, treatment was delayed due to early postoperative pain and psychological factors, causing apprehension about the potential risk of chronic pain and impediments to resuming societal activities. The primary objective was to assess the effectiveness of CBT as an intervention and its structured approach to employing CBT during the early postoperative phase.

## Case presentation

**Figure 1:**
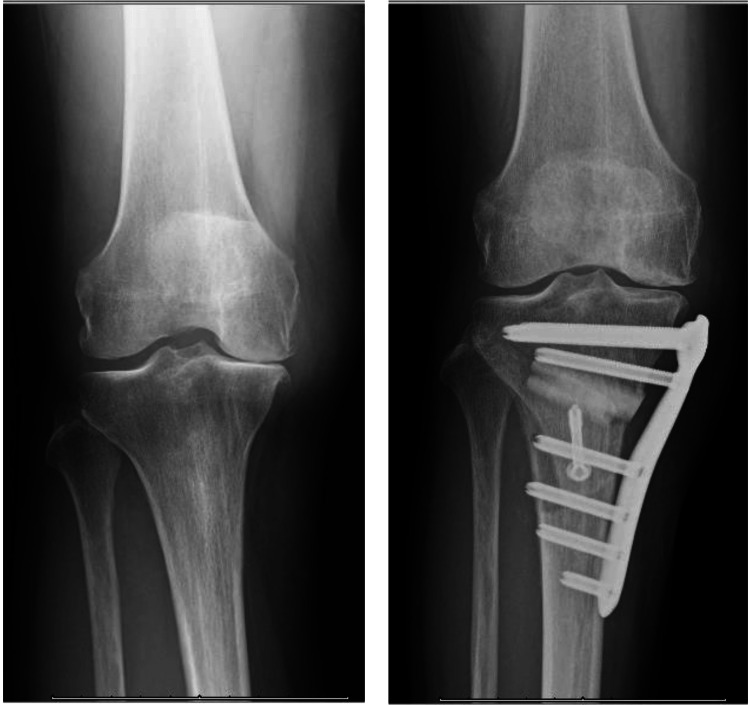
Pre- and post-operative high tibial osteotomy.

This case study involves a woman in her 60s who underwent bilateral HTO for knee pain. Five years earlier, she sought medical attention at Hospital A, where HTO was recommended and subsequently performed on her left knee, as she was deemed a suitable candidate. Her recovery from this procedure was successful, and the implant was removed the following year. However, her right knee pain worsened, necessitating admission for HTO on the right knee. Preoperatively, the right knee joint had a flexion and extension range of motion of 120° and -5°, respectively, with grade Ⅲ pathology according to the Kellgren-Lawrence classification. The surgery was performed by two orthopedic surgeons, involving an intraoperative tibial osteotomy fixed with an artificial bone plate (Figure 1).

Postoperative physical therapy began the day after surgery, with ambulation using a walker until the second week, transitioning to cane ambulation in the fourth week, with occupational therapy (OT) included as needed.

Progress to OT intervention

In the first postoperative week, the patient reported a pain score of 2/10 on the Numerical Rating Scale (NRS), with knee joint flexion and extension at 115° and -10°, respectively. By the second week, the pain score had reduced to 1-2/10, and knee flexion and extension improved to 120° and -10°, respectively. In the third week, the patient used one crutch, had a pain score of 4-5/10, and achieved 130° flexion and -10° extension in the right knee. During this time, the patient expressed anxiety about returning to work the following month. By the fourth week, the patient walked unaided with a T-cane, but the pain score increased to 8/10. Range of motion remained at 130° flexion and -10° extension, and anxiety escalated as the patient perceived slower progress than others. The Pain Catastrophizing Scale (PCS) [10] at four weeks showed a score of 26/52 (15/20 rebellion, 7/20 helplessness, and 4/12 magnification), which was higher than the two-week preoperative score of 21/52 (12/20 rebellion, 4/20 helplessness, and 5/12 magnification). Following discussions between the physical therapist and physician, the patient requested OT with CBT for pain and psychological factors in the fifth week, facilitating discharge.

Team approach

Input from the multidisciplinary team highlighted several observations related to pain and anxiety. The doctor noted an improvement in the knee condition and increased proficiency in walking with a cane. Based on these observations, it was recommended that the OT should implement CBT-based interventions. The nurse's report indicated increasing impatience, anxiety, and pain as the patient resumed work. While medication for pain management was encouraged, no significant changes were observed. The physiotherapist commented on the promising potential for knee strengthening but highlighted a peak in anxiety, especially on Sundays when rehabilitation sessions were not scheduled, unlike when other patients undergoing the same operation were discharged. The patient exhibited pessimistic thinking and anxiety, which hindered adherence to exercise therapy, including physiotherapy. It was recommended that physiotherapists should focus on exercise therapy for functional enhancement, while OTs should address psychological factors to facilitate a seamless discharge process.

OT assessment

Intake Interview and Conceptualization

**Figure 2:**
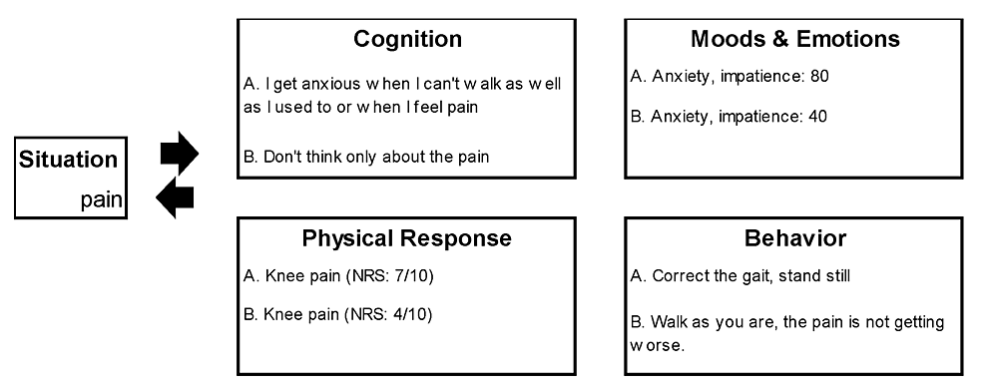
Case formulation.

At the OT intervention onset, an evaluation interview was conducted using the Canadian Occupational Performance Measure (COPM) [11] to identify the patient's needs and set goals. Long-distance walking outdoors was crucial for returning to work post-hospital discharge and was rated at 10, whereas performance and satisfaction levels were low, both at 2. During the interview, the patient expressed, "Intense pain burdens me. My knees throb, hindering household chores. I can only crawl to the bathroom." The patient acknowledged anxiety about pain and post-hospital life. Moreover, she stated, "Nothing has changed since the surgery," implying dissatisfaction despite improved gait. A conceptualization (Figure 2) revealed the patient's perception of 'pain' causing anxiety and impatience, accounting for 80% of her mood. Physical reactions included knee pain and an unsteady gait due to knee shielding. The patient adapted her walking and attempted a slower pace as a coping behavior. The patient mentioned using medications and the taught walking technique, but pain recurred after just two or three steps, increasing anxiety.

Development of OT Plan

OT practice using CBT was designed to facilitate achieving the goals outlined in the COPM. The patient's statements, such as "nothing has changed since the surgery" and "I get anxious when I feel pain," were acknowledged, recognizing that the current psychological and cognitive conditions hindered the patient's ability to engage in self-directed problem-solving and that it was not yet suitable to implement OT targeting behavioral changes. Consequently, enhancing the psychological situation was prioritized by employing cognitive restructuring, a method aimed at recognizing and modifying cognition until reality becomes clear. Upon reaching a point where addressing anxiety and reviewing the problem from a broader perspective was feasible, the intervention progressed to the second phase. During this phase, the patient acquired self-management skills, employing a coping list to handle situations involving pain and anxiety to effectively manage these symptoms. In the third phase, the OT sessions encompassed the practice of activities of daily living (ADLs), including bathing, stairway activities, and floor activities, along with instrumental ADLs such as cooking, laundry, and cleaning. The objective was to accumulate successful experiences through these actions. Furthermore, an activity diary with a focus on active pacing was introduced in this phase to prevent overactivity. Following each intervention, interviews were conducted to review problems through a structured agenda, and goals were organized and structured in manageable steps, allowing for iterative evaluations and program restructuring.

Results

Evaluation Indices

The outcomes of the evaluation indices are presented in Table 1. In the pain assessment, the NRS yielded scores of 2/10 for pain at rest, 7/10 for pain during exercise, and 8-10/10 for pain at night. The Hospital Anxiety and Depression Scale (HADS) [12] revealed scores of 13/21 for anxiety and 5/21 for depression. Self-efficacy in daily life, as assessed by the Modified Falls Efficacy Scale (MFES) [13], achieved a score of 74/140; higher scores indicate greater self-efficacy in daily living. Pain Disability Assessment Scale (PDAS) [14] was employed to gauge pain-induced life disability, yielding a score of 36/60. The PDAS measures disability from chronic pain, with higher scores indicating greater disability due to pain, with a cut-off value of 10 points. QOL was assessed using the EQ-5D [15], yielding a score of 0.279442/1, and the EQ-5D Visual Analog Scale (EQ-5D VAS) indicating 20/100 points. The number of copings was 0.

**Table 1:** Pre-OT and post-OT outcome changes. Shows the score for each outcome. OT: Occupational Therapy; COPM: Canadian Occupational Performance Measure; NRS: Numeric Rating Scale; PCS: Pain Catastrophizing Scale; HADS: Hospital Anxiety and Depression Scale; MFES: Modified Falls Efficacy Scale; PDAS: Pain Disability Assessment Scale; FIM: Functional Independence Measure; EQ-5D-5L: EuroQol 5 Dimensions 5-Level; VAS: Visual Analog Scale.

	Pre-OT	Post-OT
COPM-performance	2	6
COPM-satisfaction	2	6
NRS	7	4
PCS	26	13
HADS depression	5	5
HADS anxiety	13	0
MFES	74	113
PDAS	36	13
FIM™	119	125
EQ-5D	0.279442	0.667149
EQ-5D VAS	20	60
Coping skill (number)	20	0

Occupational Therapy Progress

Phase 1: The cognitive reconstruction method (Table 2) was implemented to recognize and modify cognition to facilitate behavioral changes. This method entails focusing on the automatic thoughts that arise during highly stressful situations and, by clarifying cognitive distortions, fostering awareness of adaptive thinking.

**Table 2:** Cognitive reconstruction.

Number	Session	Case description
1)	Situation	Pain when walking
2)	Mood (%)	Anxiety, impatience: 80%
3)	Cognition	I may not be able to walk again
4)	Rationale	Pain was the same as before surgery, leg condition was the same, opposite knee was progressing wel
			5)	Refutation	I can walk with a cane, this knee was worse before surgery
						6)	Adaptive thinking	II can walk with a cane better than before the surgery, the knee was worse from the beginning, I was going to live even if it hurt before the surgery
7)	Current mood (%)	Anxiety, impatience: 60						

In the cognitive restructuring process, the patient exhibited anxiety and impatience concerning pain while walking and the perception that they may no longer be able to walk. The patient's reasoning for this apprehension was that the pain remained unchanged from preoperatively, the knee condition had not improved, and the procedure was more effective when performed on the opposite leg. However, upon presenting counterarguments, the patient acknowledged, "I am capable of walking with a cane. The leg I operated on this time was worse before the surgery." Furthermore, the patient recalled, "Before the surgery, I was limping, yet I still went out as I pleased." Consequently, adaptive thinking encompassed statements such as, "Previously, I had a limp, but now I can walk with a cane without a limp. The opposite knee responded well, while the knee in question was already problematic. Despite the pain, I managed to do what I desired before the surgery. There is no time for me to be disheartened at present." Following the cognitive restructuring intervention, anxiety and impatience diminished by 60%. Additionally, upon reviewing pictures of herself engaged in normal activities and contrasting them with her current condition, the patient noted, "Before and after the surgery, my legs appear straight, and I do not stand in a manner suggestive of pain." Embracing a more positive outlook, the patient expressed, "Things are improving." The patient's newfound positive outlook was further evident in statements such as, "I feel as if I have regained my true self today" or "It feels like I haven't laughed in a long time." Based on these observations, it was determined that the patient was now capable of interpreting reality through cognitive restructuring and engaging in self-directed problem-solving. Thus, the progression to the self-management phase was recommended.

Phase 2: Self-management using coping lists [16] was implemented to foster coping skills in situations of pain and anxiety. This process, in line with the principles advocated by Folkman S and Lazarus RS [17], encompasses the evaluation of stressful circumstances, incorporating primary and secondary assessments and responses to stressors. To promote self-understanding, the patient's pain situation was reviewed chronologically through interviews, organizing the timing of pain occurrences and the ensuing anxiety. Patient activities and cognition during pain-free periods were externalized, and appropriate coping behaviors were discussed objectively. Drawing from the patient's preoperative hobbies and preferences, coping skills that were predicted to be effective for the patient were complied with. Notably, the patient experienced anxiety when alone on Sunday nights after rehabilitation and after walking to collect hot water. As a result, strategies to organize her thought processes through appropriate coping behaviors were examined. The identified effective coping skills included not only physical activities such as icing and medication but also engagement in activities such as listening to music and watching movies. Furthermore, the patient demonstrated an improvement in pain management behavior through interactions with other patients, notably through discussing her pain with them. On Sundays after the intervention, the patient engaged in voluntary training, physical therapy, and conversations with roommates and made phone calls to the patient's office. The patient expressed, "Yesterday, I was occupied with numerous activities, and time passed quickly. Nevertheless, I still feel anxious because the person I spoke to Yesterday won't be here next Sunday." Despite lingering anxiety, the patient reflected positively on the fact that she managed to get through Sundays. Additionally, the patient recognized a shift in outlook, stating, "The pain will never disappear entirely, but I am learning to cope with it better." The patient employed direct measures such as icing, relaxation, and mindfulness techniques to address nocturnal pain. Six days after the intervention, the patient remarked, "I still experience pain, but it has improved compared to before. I sense a slight difference; it's like I am trying to manage it by taking painkillers. I understand that the pain won't change unless I confront it head-on." The patient displayed positive comments regarding pain management. Given the patient's specific desire for discharge, consent was sought and obtained to proceed with implementing actual behavioral changes.

**Figure 3:**
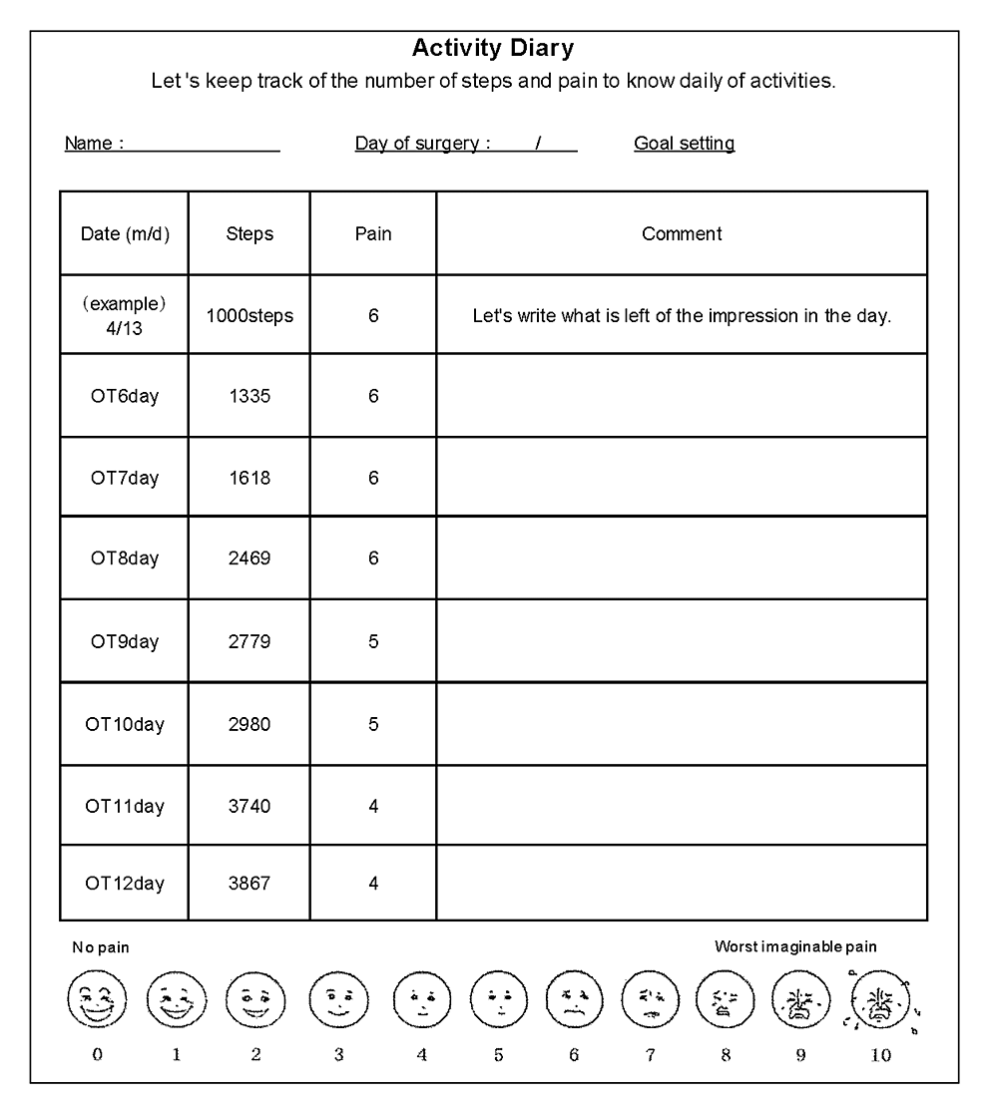
Activity diary.

Phase 3: Behavior change through OT, active pacing (Figure 3): Once the patient acquired OT self-management skills, behavioral changes were implemented to facilitate her discharge from the hospital. Active pacing was employed through the use of an activity diary and practical movement training. Initially, ADLs such as bathing, stair navigation, and floor movements, along with instrumental ADLs like cooking, laundry, and cleaning, were practiced step-by-step as part of actual OT interventions. As a result, the patient was able to perform these activities without experiencing anxiety.

Subsequently, an activity diary [18] was introduced to promote appropriate behavioral change aimed at preventing pain deterioration. Throughout the diary, the patient gradually improved activity levels without exacerbating pain. During the case study, the patient articulated, "I feel anxious and fearful about leaving the hospital, but it is not beneficial to be consumed by pain. I will proceed gradually and independently." These positive remarks were indicative of the patient's proactive mindset. Following the final interview, OT was concluded upon the patient's discharge from the hospital, as the patient demonstrated successful adaptation to the new behavioral changes and improved pain management.

OT final evaluation (Table 1): The COPM in both performance and satisfaction improved to 6. The NRS was reduced for pain at rest to 0/10 and pain during movement to 4/10. The PCS results were rebellion, 3/20; helplessness, 9/20; and magnification, 0/12, totaling an improvement to 13/52. The HADS results revealed an anxiety score of 0/21 and a depression score of 5/21, indicating reduced catastrophic thinking, anxiety, and depression. The PDAS was 13/60, MFES was 113/140, and self-efficacy and life disability improved. QOL also improved; EQ-5D was 0.667149/1, EQ-5D VAS was 60/100, and the number of copings was 20. In the CBT conceptualization, the patient's mood improved to 40% for anxiety and impatience due to the perception that her pain situation was "getting better little by little," her physical response was knee pain, and her behavior was to continue walking at her own pace. The patient's anxiety was regarding discharge from the hospital and returning to work.

## Discussion

The most important finding of the study is that the combination of OT and CBT holds the potential to enable a successful discharge home. Individuals can gain the tools to effectively self-manage anxiety and pain in the postoperative phase through an individualized intervention emphasizing non-pharmacological self-management. The findings indicate that CBT may be a valuable approach for enhancing psychological factors associated with pain, mitigating the risk for chronic pain development.

Cognitive restructuring method

In scenarios concerning individuals afflicted by intense pain and distress, the execution of proposed alterations in behavior presents a formidable endeavor. Henceforth, it is essential to mitigate psychological manifestations by facilitating the patient's attainment of a more astute apprehension of reality via the cognitive reconfiguration methodology, which aims to identify and amend cognitive paradigms. Previous investigations have exhibited the efficacy of CBT, inaugurated by cognitive reconfiguration, in improving psychological constituents such as catastrophic ideation in subjects grappling with persistent pain [19]. Thus, the employment of cognitive restructuring with the aim of behavioral change could possibly have facilitated a triumphant progression toward the coveted behavioral adaptation.

Coping skills

In the process of goal setting and reorganizing thoughts through cognitive restructuring, it became essential to acquire coping skills for effective self-management of anxiety and pain. Riddle DL et al. [20] reported that practicing temporary coping with pain, self-relaxation, and coping skills to manage activities while considering pain enhanced psychological well-being and functional abilities in patients with musculoskeletal disorders. They also observed that implementing coping skills in early postoperative patients resulted in effective self-management of pain and anxiety, ultimately facilitating successful goal achievement [16]. In our case, it is evident that the patient was able to effectively manage pain and anxiety by actively engaging in efforts to improve pain and anxiety using coping skills following cognitive restructuring.

Behavior change and activity diary toward goal achievement

Upon achieving self-management of pain and anxiety, the focus shifted to implementing practices aimed at behavioral changes in pursuit of the established goals. Previous studies have demonstrated improvements in life disability and QOL in chronic pain patients through CBT, which facilitates behavioral changes after cognitive modifications [19]. Moreover, in Japan, during the early post-total knee replacement period, target activities are set for patients, and goal attainment is employed, while pain and step count are tracked using an activity diary, which is expected to enhance pain management, address psychological factors like catastrophic thinking, and improve activity levels [18]. In the present case, we believe that, in addition to facilitating behavioral changes toward the established goals, the introduction of an activity diary as a suitable behavioral activation tool further enabled the implementation of step-by-step behavioral changes.

Limitations

However, this research has some limitations that need acknowledgment. Firstly, it is crucial to understand that CBT is not universally applicable in the early postoperative period and necessitates an individualized approach, dependent on evaluations from other healthcare professionals and occupational therapists who are primary care providers. For instance, it can be especially pertinent for patients exhibiting psychological risk factors predisposing them to chronic pain. To better evaluate its efficacy, future studies should involve larger sample sizes and morph into intervention studies. Crucially, to measure the sustained impact of integrating OT and CBT practices during the early postoperative phase, it is essential to conduct long-term assessments. Additionally, the establishment of a robust infrastructure for CBT is vital in Asian countries, with Japan pioneering to facilitate its development and application.

## Conclusions

This study introduces the application of OT in conjunction with CBT during the early postoperative period for cases characterized by psychological factors and reduced QOL due to pain. These findings align with previous Japanese studies on CBT for chronic pain patients, demonstrating its effectiveness in addressing psychological factors. In nations like Japan, where manual and traditional therapies are commonly favored for individuals with chronic pain, we propose that the implementation of CBT during the early postoperative stage, prior to the development of chronic pain, combined with its integration into self-management through cognitive and behavioral techniques addressing pain and psychological aspects, could mitigate the risk of chronic pain development. Consequently, this approach can potentially facilitate lasting postoperative societal reintegration and enhance overall QOL. This study postulated that meaningful enhancements in long-term postoperative societal reintegration and overall QOL are achievable.
